# Iron regulatory protein 1-deficient mice exhibit hypospermatogenesis

**DOI:** 10.1016/j.jbc.2024.108067

**Published:** 2024-12-10

**Authors:** Aileen Harrer, Niraj Ghatpande, Tiziana Grimaldini, Daniela Fietz, Vishnu Kumar, Christiane Pleuger, Monika Fijak, Dankward T. Föppl, Lennart P. Rynio, Hans-Christian Schuppe, Adrian Pilatz, Marek Bartkuhn, Tara Procida-Kowalski, Noga Guttmann-Raviv, Sudhanshu Bhushan, Esther G. Meyron-Holtz, Andreas Meinhardt

**Affiliations:** 1 Institute of Anatomy and Cell Biology, Unit of Reproductive Biology, Justus-Liebig-University of Giessen, Giessen, Germany; 2 Hessian Centre of Reproductive Medicine, Justus-Liebig-University Giessen, Giessen, Germany; 3 Faculty of Biotechnology and Food Engineering, Technion-Israel Institute of Technology, Technion City, Haifa, Israel; 4 Institute for Veterinary Anatomy, Histology and Embryology, Justus-Liebig-University of Giessen, Giessen, Germany; 5 Department of Urology, Pediatric Urology and Andrology, Justus-Liebig-University of Giessen, Giessen, Germany; 6 Biomedical Informatics and Systems Medicine, Science Unit for Basic and Clinical Medicine, Justus-Liebig-University of Giessen, Giessen, Germany

**Keywords:** testis, spermatogenesis, IRP1, iron homeostasis, reactive oxygen species (ROS), DNA damage, DNA repair

## Abstract

Imbalances in testicular iron levels are linked to compromised sperm production and male infertility. Iron regulatory proteins (IRP) 1 and 2 play crucial roles in cellular iron regulation. We investigated the role of IRP1 on spermatogenesis using *Irp*1-deficient mice (*Irp*1^−/−^). Histological analysis of the testis of *Irp*1^−/−^ mice revealed hypospermatogenesis with a significant reduction in the number of elongated spermatids and daily sperm production compared to wild-type (WT) mice. Flow cytometry of germ cells from WT and *Irp*1^−/−^ mice showed reduction in spermatocytes and round and elongated spermatids in *Irp*1^−/−^ mice, which was confirmed by histological and immunofluorescence quantification. Finally, stage VIII of spermatogenesis, crucial for spermatid maturation, was less frequent in *Irp*1^−/−^ testicular cross-sections. Hypospermatogenesis worsened with age despite unchanged intratesticular iron levels. Mechanistically, this was due to increased oxidative stress indicated by elevated 8-Oxoguanine (8-OxoG) levels, a DNA lesion resulting from reactive oxygen species (ROS). Furthermore, bulk RNA-seq data indicated compromised DNA damage repair and cell cycle processes, including mitosis and meiosis in *Irp*1^−/−^ mice, which may explain hypospermatogenesis. Our results suggest that IRP1 deletion leads to hypospermatogenesis due to impaired cell cycle progression, decreased DNA damage repair capacity, and oxidative damage. Altogether, this study uncovers a role for IRP1, independent of traditional mechanisms of iron regulation.

Infertility is a multifaceted condition, affecting approximately one in seven couples in Western countries, with the male factors contributing to nearly 50% of cases, figures projected to increase (1, 2, 3). Studies in patients suffering infertility have shown that common conditions detected after bilateral biopsy of the testes are Sertoli cell-only syndrome (around 25–37%), hypospermatogenesis (approximately 19%), and germ cell maturation arrest (approximately 7–12.5%) (4, 5). Although all germ cell types are present in hypospermatogenesis, their numbers are reduced compared to normal. The most noticeable reduction is often seen in the elongating spermatids, which are the most mature germ cells in spermatogenesis. The decreased number of elongating spermatids typically results from a reduction in the number of dividing precursor cells (spermatogonia, spermatocytes). With each of them generating four spermatids, compromising the initial stages of spermatogenesis leads to a potentiated negative effect in the more advanced stages of development.

Spermatogenesis can be divided into four critical phases (1): the mitotic division of the spermatogonial stem cells (2), meiotic divisions of primary and secondary spermatocytes to reduce the chromosome set (3), cellular transformation of haploid germ cells (round spermatids) into elongated spermatids, and (4) release of spermatozoa from the seminiferous epithelium into the tubular lumen (6, 7, 8, 9). These processes are highly sensitive to oxidative stress and iron fluctuation (10, 11).

Iron is essential for biological processes including DNA replication, mitochondrial function, and redox homeostasis (10). Iron homeostasis is tightly regulated both systemically and cellularly. On the systemic level, the peptide hormone hepcidin controls cellular iron export through ferroportin, while cellularly, iron regulatory proteins 1 (IRP1/ACO1) and IRP2/IREB2 play major roles in maintaining iron balance (12, 13). Targeted deletion of IRP2 leads to a systemic disruption of iron metabolism, underscoring the dominant role of IRP2 in the regulation of iron homeostasis (14). In contrast, IRP1 deletion impairs iron metabolism in a transient and tissue-specific manner (15).

IRP1 is a bifunctional protein whose role changes with cellular oxygen and iron levels (16). Under normal conditions, IRP1 forms a [4Fe-4S]-cluster, functioning as an aconitase. Under iron deficiency and in the presence of sufficient oxygen, or elevated ROS, IRP1 loses this cluster and binds to iron-responsive elements (IREs) in mRNAs of target genes (17). The high stability of its Fe-S cluster at physiologic oxygen levels suggests it integrates cellular oxygen and ROS sensing with iron status.

Studies in rats have shown that increased iron concentration leads to oxidative DNA damage, reduced testicular antioxidants, and impaired sperm counts and motility (11, 18). Similarly, human studies have linked imbalances in testicular iron levels to compromised sperm production and male infertility. Thus, maintenance of iron homeostasis within the testicular microenvironment is crucial for male reproductive health (19).

The role of iron regulatory proteins (IRPs) in male reproductive organs, particularly in spermatogenesis, remains unexplored. Considering the tissue-specific role of IRP1 in iron metabolism and the importance of controlled iron homeostasis for testicular function, this study investigates the effect of IRP1 deletion on mouse spermatogenesis.

## Results

### IRP1 deletion causes hypospermatogenesis and low daily sperm production

**Figure 1:**
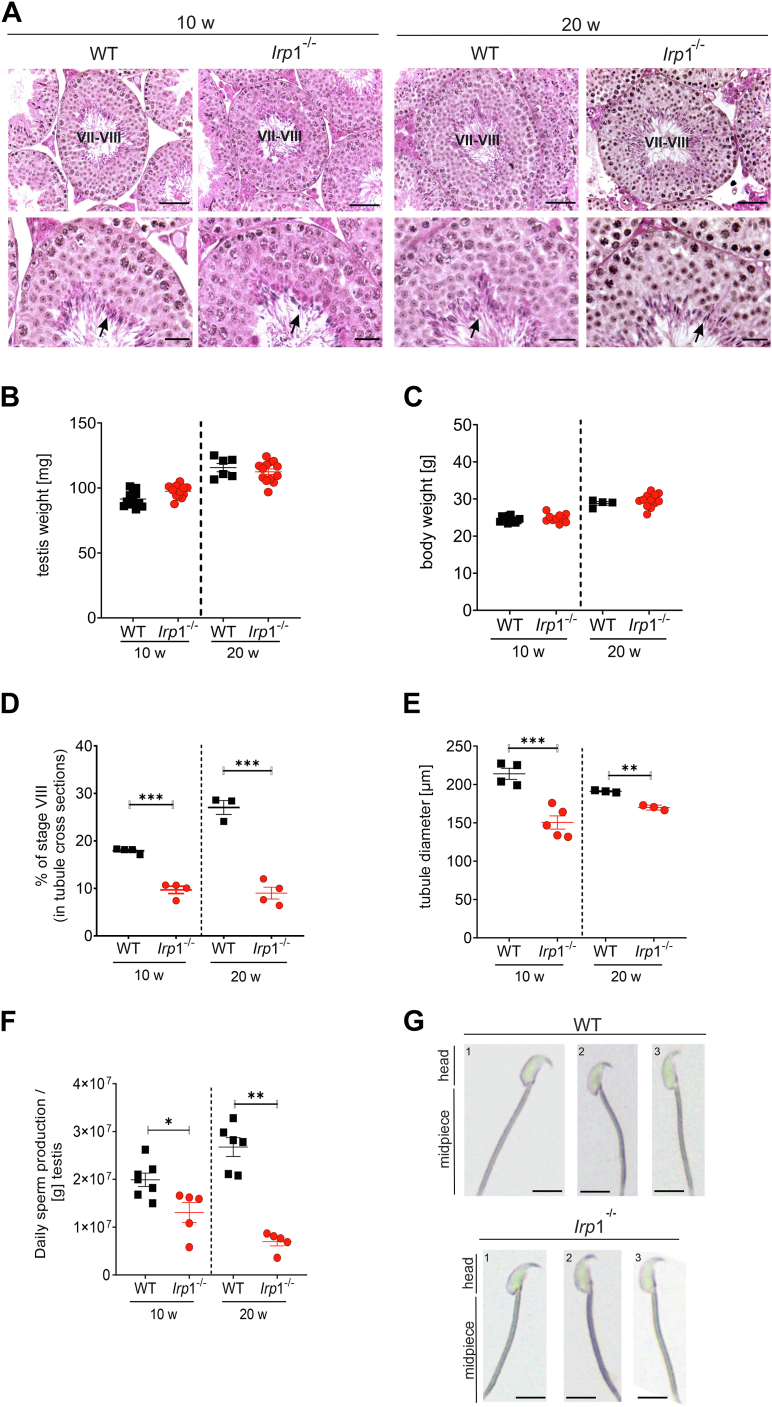
**Deletion of IRP1 in testis shows hypospermatogenesis in mice.***A*, hematoxylin and eosin (H&E) staining of paraffin testis sections from wild-type (WT) and *Irp1*^*−/−*^ mice at 10 and 20 weeks of age. Representative images of stage VII-VIII of spermatogenesis are shown (scale bar: 50 μm for the overview, 20 μm for the higher magnification). *Black arrow* = elongated spermatids prior to spermiation (n = 5 mice per group). *B* and *C*, testis and body weights of 10- and 20-week-old mice (n = 10–20 mice per group). Testis weights represent the mean of left and right testes of each animal (n = 10–20 mice per group). Data are presented as dot plots. *D*, quantification of seminiferous tubules undergoing spermiation (Stage VIII) in testicular cross-section of 10- and 20-week-old mice. Only tubules with a round to slightly ovoid appearance were assessed. Data are presented as a dot plot (n = 3–4 mice per group). *E*, diameters of 100 round seminiferous tubules with a round to slightly ovoid appearance were measured from 10-week-old mice (n = 3–5 mice per group). *F*, daily sperm production (DSP) per gram (g) testis at 10 and 20 weeks of age. *G*, sperm morphology was assessed by sperm swim-out assay from the cauda epididymis followed by light microscopy evaluation. Representative images are shown (scale bar: 10 μm; n = 6 mice per group). Statistical significance was determined by unpaired student's *t* test (∗*p* < 0.05, ∗∗*p* < 0.01, ∗∗∗*p* < 0.001).

Hematoxylin and eosin (H&E) stained testicular cross-sections of *Irp*1^−/−^ mice showed reduced numbers of elongating spermatids at stage VIII of spermatogenesis compared to WT in both 10 and 20-week-old mice, indicating hypospermatogenesis (Fig. 1*A*). Testicular and body weights and their ratio were comparable, yet stage VIII of spermatogenesis was underrepresented in histological specimens, and the diameter of seminiferous tubules was reduced in both age groups (Fig. 1, *B*–*E*). Histological observations were corroborated by a significant reduction in daily sperm production (DSP) in *Irp1*^*−/−*^ mice at 10 weeks of age, which was further decreased at 20 weeks compared to age-matched WT animals (Fig. 1*F*). Despite these observations, sperm morphology did not differ between genotypes (Fig. 1*G*) and normal litter sizes (data not shown) in *Irp1*^*−/−*^ breeding pairs indicate that sperm is functional and sufficient for fertilization.

### Significant reduction in spermatocytes in *Irp*1^−/−^ testis

**Figure 2:**
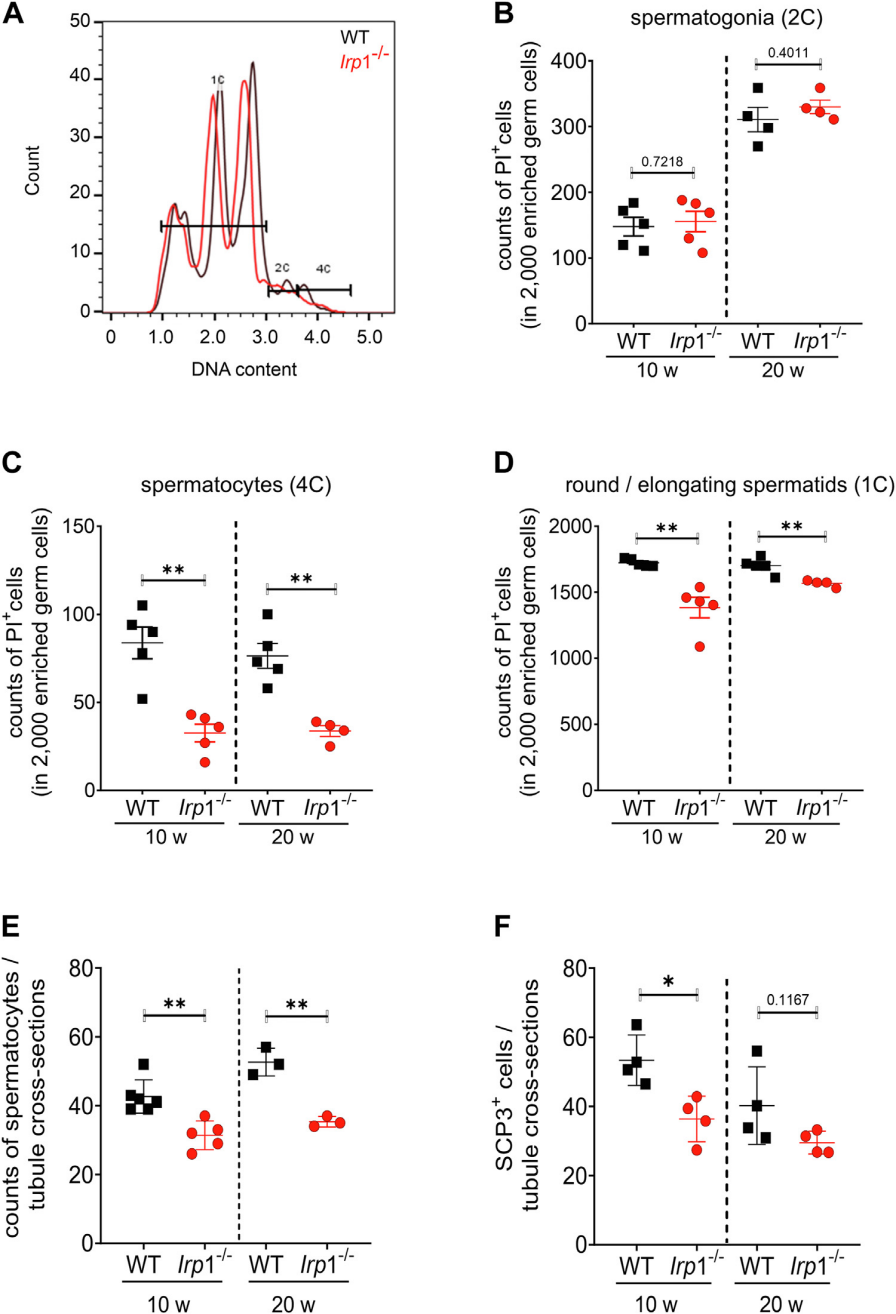
**Germ cell numbers are reduced in *Irp*1**^**−/−**^**mice.***A*, flow cytometry analysis was performed on germ cell enriched cell suspensions to assess cells of different DNA content by propidium iodide (PI) staining within the germ cell population (2000 cells analyzed). Cells were distinguished as follows: 2C (spermatogonia and pre-leptotene (S-phase)), 4C (primary spermatocytes), and 1C (round and elongating spermatids). *B–D*, the differentially categorized germ cells were quantified (*B*) 2C (spermatogonia), (*C*) 4C (primary spermatocytes), and (*D*) 1C (round and elongating spermatids). Data are presented as dot plots (n = 4–5 mice per group). *E* and *F*, the abundance of primary spermatocytes was determined using two complementary methods: (*E*) H&E staining of testicular sections to count primary (pachytene) spermatocytes based on morphological characteristics. *F*, quantification of immunofluorescence staining of SCP3^+^ cells on testis sections from *Irp1*^*−/−*^ and WT mice. Only round to slightly ovoid seminiferous tubules were analyzed in five randomly selected images per group (n = 3–6 mice per group). Statistical significance was determined by unpaired student's *t* test (∗*p* < 0.05, ∗∗*p* < 0.01, ∗∗∗*p* < 0.001).

Hypospermatogenesis is characterized by a reduced number of germ cells within the seminiferous tubules of the testis. To identify which germ cell population was affected, we performed DNA staining followed by flow cytometric analysis. Propidium iodide staining revealed a significant decrease in total germ cell numbers in *Irp*1^−/−^ testes compared to WT in both age groups (Fig. 2, *A*–*D*). This reduction specifically affected spermatocytes, round and elongating spermatids, and spermatozoa, but not spermatogonia (Fig. 2*B*), indicating that *Irp1* deficiency impacts multiple steps of spermatogenesis, but not the stem cell niche within the seminiferous epithelium (Fig. 2*B*). The haploid round and elongated spermatids (1C) (Fig. 2*D*) and the tetraploid primary spermatocytes (4C DNA containing cell population, Fig. 2*C*) were both reduced in 10- and 20-week-old *Irp*1^−/−^ testes compared to WT, while the spermatogonia (2C) were at best mildly reduced (Fig. 2*B*). Flow cytometry data were further validated by quantitative determination of primary spermatocytes based on morphological criteria (cell size, chromatin structure) in hematoxylin and eosin-stained testicular sections. These showed a significant reduction in 10-week-old*-Irp*1^−/−^ testes compared to WT, with a trend toward reduction in 20-week-old mice (Fig. 2*E*). These results were confirmed by quantification of immunofluorescence detection of synaptonemal complex protein 3 (SCP3), a marker for primary spermatocytes (Fig. 2*F*). Together these data suggest that IRP1 deficiency impairs mitotic and/or meiotic divisions in germ cell development.

### Whole transcriptomic expression analysis shows alterations in genes related to cell cycle regulation, DNA repair, and oxidative stress response in *Irp*1^*−/−*^ testis

**Figure 3:**
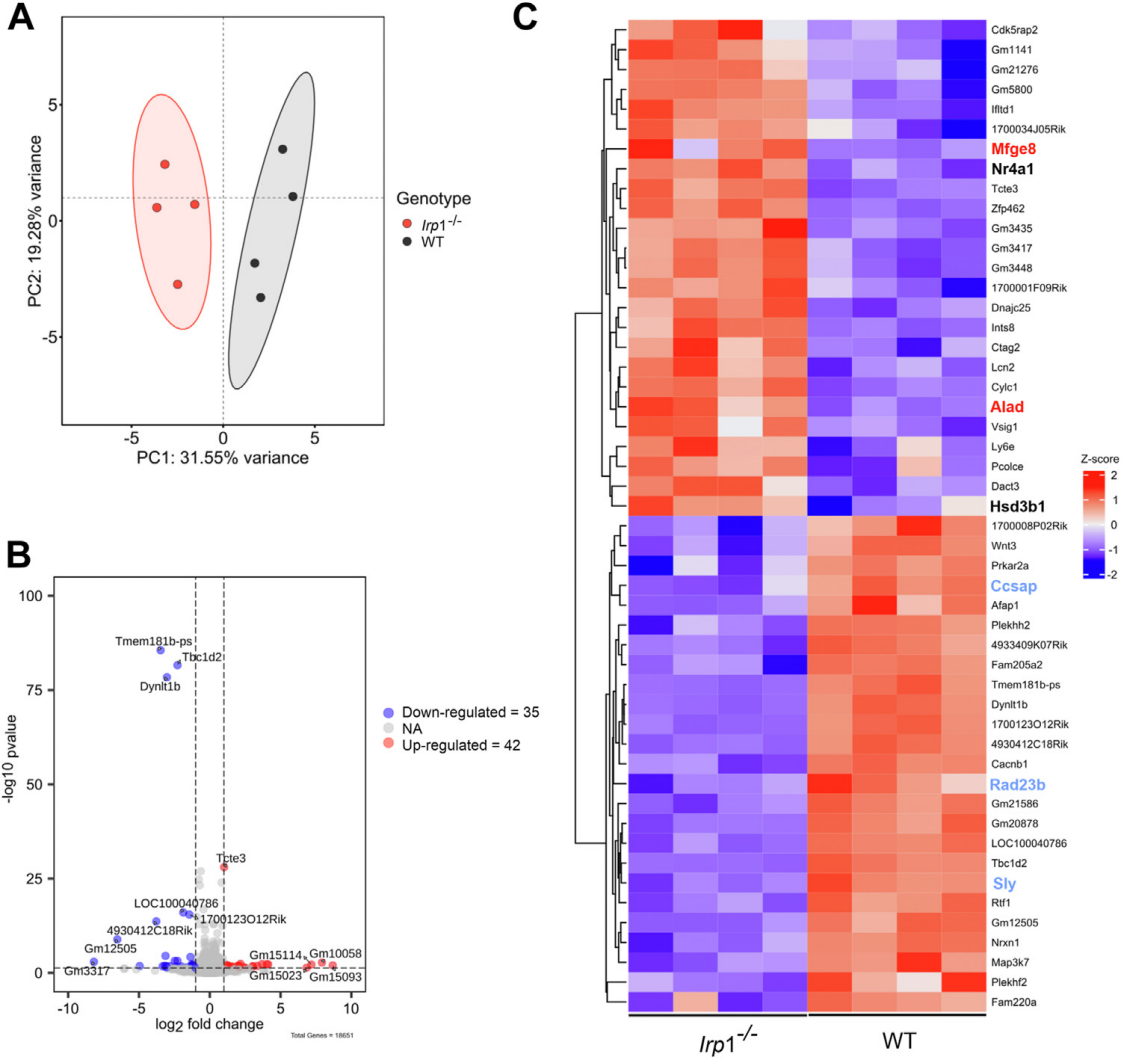
**Whole transcriptomic analysis of differential gene expression in *Irp*1**^**−/−**^**and WT mouse testis.** The examination of transcriptional changes of testicular RNA was performed for 20-week-old WT and *Irp*1^−/−^ mice. *A*, principal component analysis (PCA) demonstrates distinct clustering of WT (*black*) and *Irp*1^−/−^ (*red*) data sets. *B*, a volcano plot visualizes the differentially expressed genes (DEGs) between *Irp*1^−/−^ and WT. Using cut-off settings of log2FoldChange >1 and padj< 0.05 only 35 down-regulated and 42 up-regulated genes were identified as differentially expressed. *C*, a heatmap displays the expression patterns of the top 50 up- and downregulated genes in *Irp*1^−/−^ and WT testis. Genes related to oxidative stress (*red bold* characters), steroid biosynthesis (*black bold* characters), cell cycle and DNA repair (*blue bold* characters) are highlighted, respectively.

**Figure 4:**
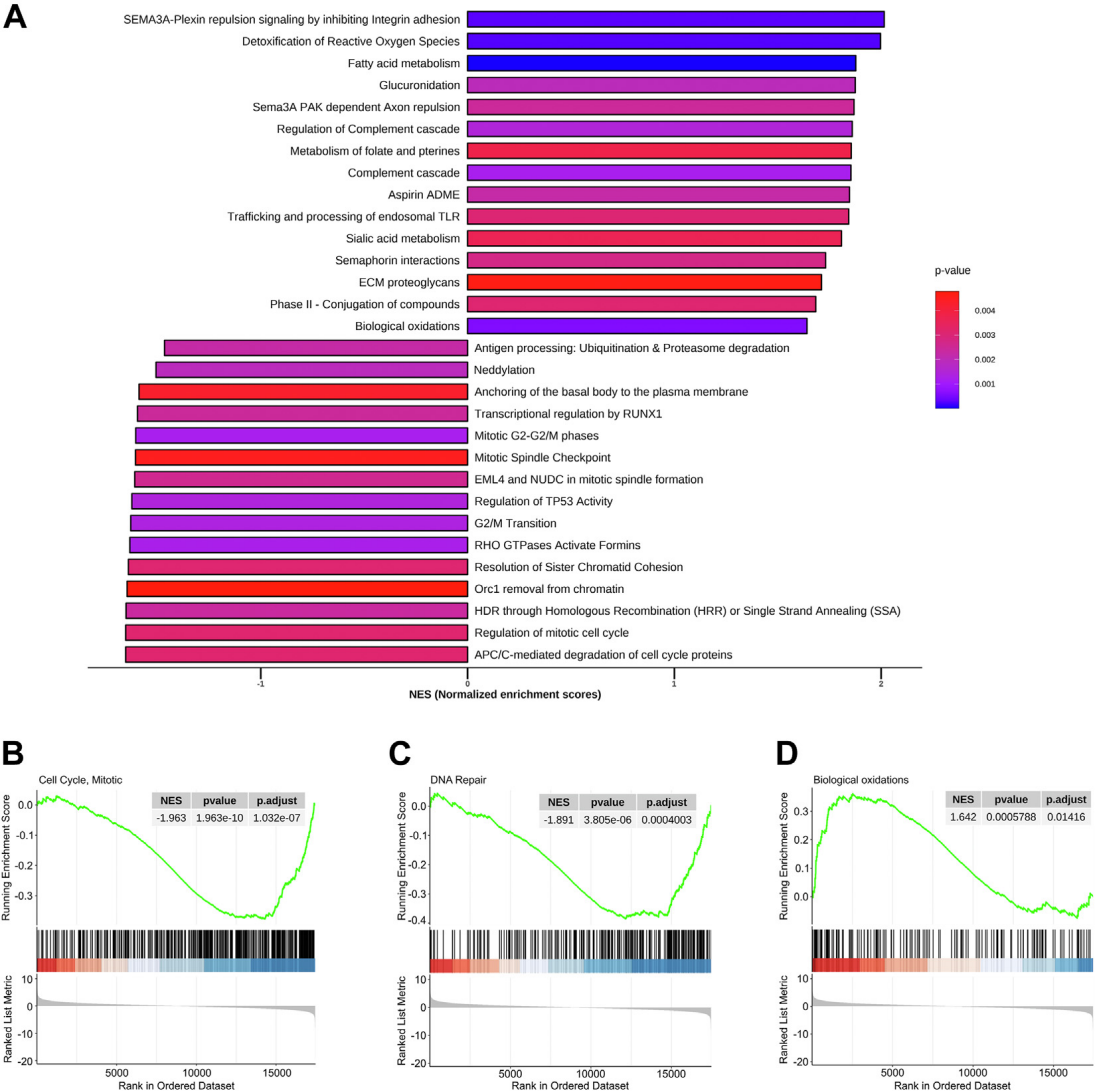
**Gene set enrichment analysis (GSEA) shows up- and downregulated pathways in *Irp*1**^**−/−**^***vs.* WT testes.***A*, bar diagram shows the GSEA (Gene Set Enrichment Analysis) results of Reactome pathways. The pathways were sorted by absolute values of the Normalized Enrichment Score (NES) and grouped by the sign of NES. The bar diagram represents 30 up- and down-regulated pathways. *B*, enrichment plots reveal negative enrichment of “cell cycle, mitotic” and (*C*) “DNA repair” pathways. *D*, positive enrichment for the “biological oxidations” pathway.

To understand the molecular causes of impaired germ cell development in *Irp1*^*−/−*^ testis, we examined changes in the whole testicular transcriptome by bulk RNA sequencing (bulk RNA-seq) in 20-week-old mice. The principal component analysis demonstrated a clear distinction between *Irp*1^−/−^ and WT testicular transcriptomes (Fig. 3*A*). In total, 19,004 genes were found to be differently expressed (Fig. 3*B*), whereby the top 50 up- and downregulated genes included several transcripts related to oxidative stress response such as delta-aminolevulinate dehydratase (*Alad*) (Fig. 3*C*), and genes regulating cell cycle and DNA repairs, such as centriole, cilia and spindle associated protein (*Ccsap*) and RAD23 homolog B, nucleotide excision repair protein (*Rad23b*). Gene set enrichment analysis (GSEA) indicated downregulation of pathways related to the cell cycle (*e.g.* “cell cycle mitotic” (Normalized Enrichment Score NES: −1.96356156, Fig. 4*B*) and “DNA-repair” (NES: −1.891545, Fig. 4*C*). Upregulated pathways included “biological oxidation” (NES: 1.642, Fig. 4*D*) and metabolic pathways for fatty acids (Fig. 4*A*). These findings suggest that germ cell loss in *Irp1*^*−/−*^ testes is potentially linked to increased oxidative stress, reduced DNA repair capacity, and impaired cell cycle regulation.

### Unchanged iron status in *Irp*1^−/−^ testis

**Figure 5:**
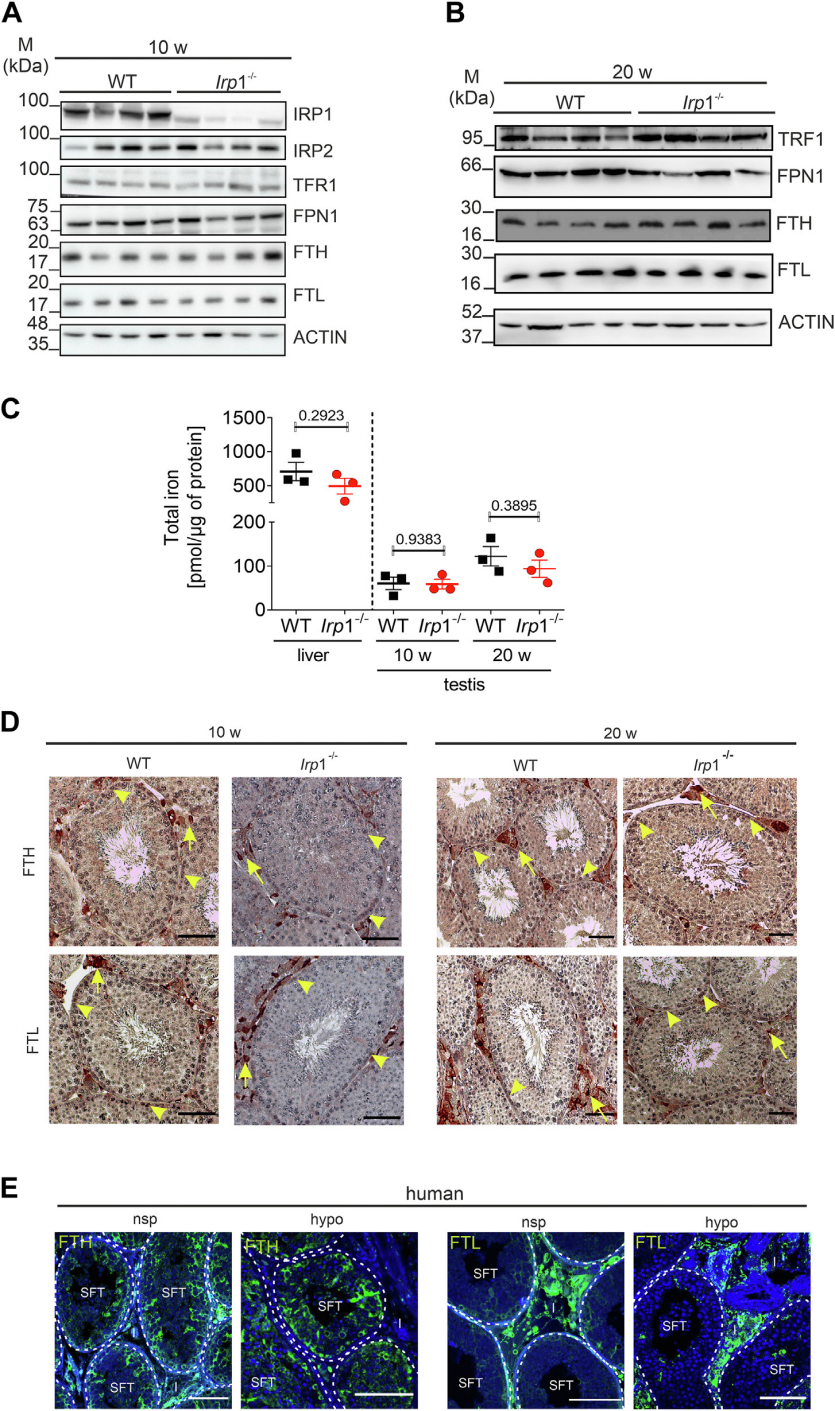
**No changes in testicular iron levels in *Irp1***^***-/***^***mice*.***A* and *B*, Western blot analysis of testicular protein extracts of WT and *Irp1*^*−/−*^ mice at 10 and 20 weeks of age. Representative blots are shown (n = 4 mice per genotype). *C*, total iron concentrations in testicular lysates from 10- and 20-week-old WT and *Irp1*^*−/−*^ mice were measured using the Ferrozine assay. Data is presented as a dot plot graph (n = 3 mice per group). *D*, immunohistochemical staining on 5 μm sections of testis from 10- and 20-week-old WT and *Irp1*^*−/−*^ mice was used to localize the distribution of ferritin-H (FTH) and ferritin-L (FTL). Representative micrographs are shown. *Yellow arrows* indicate Ferritin-positive interstitial cells (such as macrophages) and *yellowarrowheads* point to ferritin-positive germ cells (spermatogonia) (scale bar: 30 μm, n = 5 mice per group). *E*, human testicular biopsies with normal spermatogenesis (nsp) and mild hypospermatogenesis (hypo) were stained for FTH and FTL. Representative images are provided (scale bar: 100 μm, n = 3 biopsies from individual patients). Statistical significance was determined using the unpaired student's *t* test (ns > 0.05) FPN1, ferroportin 1; FTH, ferritin-H; FTL, ferritin-L; I, interstitial space; IRP1, iron-regulatory protein; IRP2, iron-regulatory protein 2; SFT, seminiferous tubule; TRF1, transferrin-receptor 1.

Given the role of IRP1 in iron metabolism, we examined potential changes in iron levels and iron homeostasis proteins. No significant differences were observed in the expression levels of ferritin-H, ferritin-L, ferroportin, transferrin receptor 1, IRP2, nor the spatial distribution of ferritin H + L between WT and *Irp*1^−/−^ testis across ages (Fig. 5, *A*, *B*, and *D*). Total iron concentrations were also unaltered (Fig. 5*C*). Immunohistochemistry revealed that both ferritin-H and ferritin-L were primarily located within the interstitial space, most likely in Leydig cells and macrophages as numbers of positive cells and niche indicate. In addition, ferritin was detected in spermatogonia at the base of the seminiferous tubules (Fig. 5*D*). Biopsies from human testes with normal spermatogenesis and hypospermatogenesis showed a similar distribution pattern of FTL and FTH, and in addition, ferritin-H positive Sertoli cells in the seminiferous epithelium (Fig. 5*E*). Further IRP1 staining of testicular sections from human and mouse sections reveals that IRP1 is mainly located in Sertoli cells, Leydig cells, and germ cells. These data were confirmed using published single-cell RNA seq data sets of human and mouse testis ((20, 21), data not shown). These findings indicate that hypospermatogenesis in *Irp*1^−/−^ mice, and likely in humans, arises from mechanisms independent of gross iron imbalance.

### IRP1 deletion in testis increases susceptibility to oxidative stress

**Figure 6:**
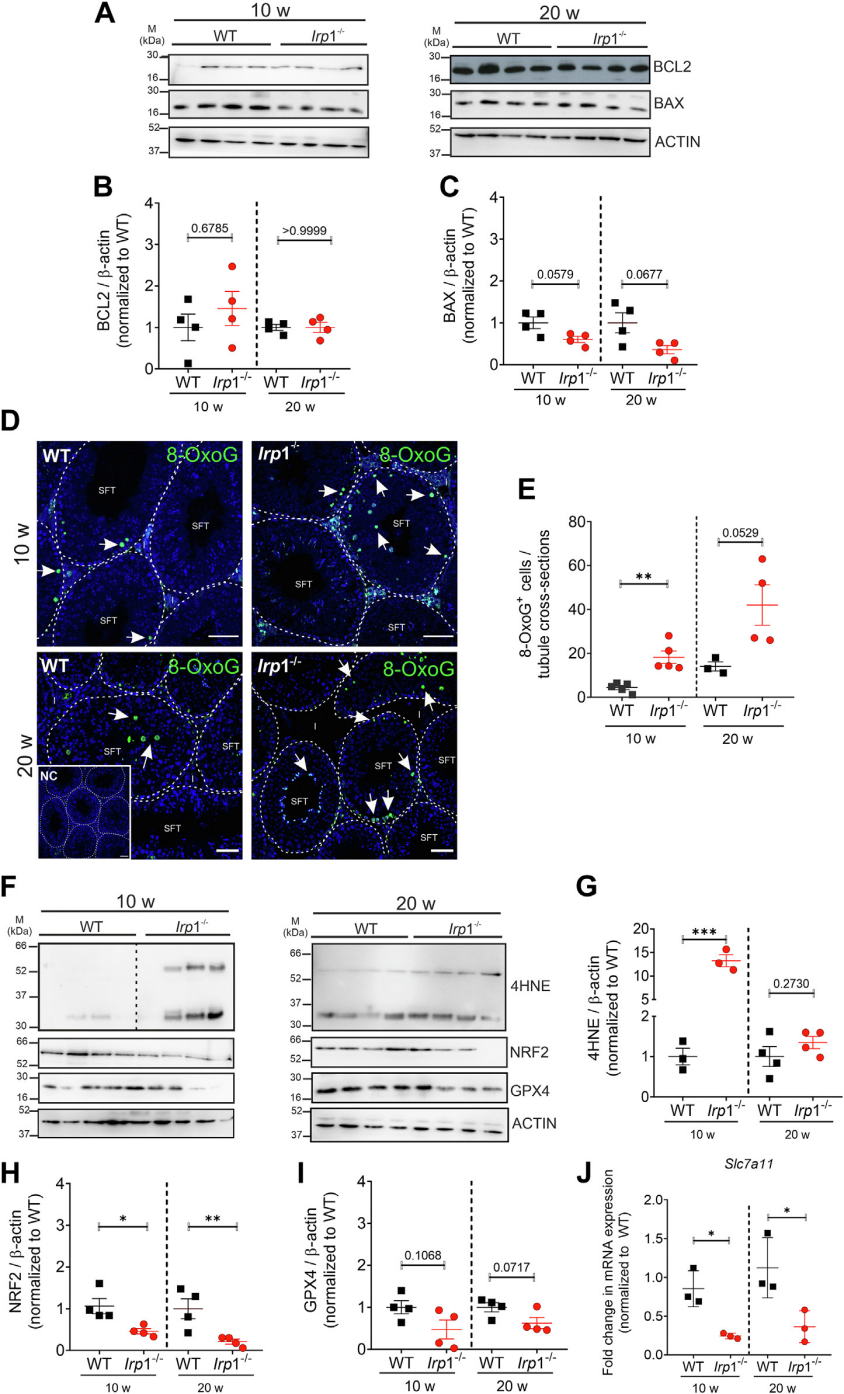
***Irp1***^***−/−***^**testis shows increased oxidative damage.***A* and *D*, Western blot analysis was conducted on lysates prepared from testis of WT and *Irp1*^*−/−*^ mice aged 10 and 20 weeks. Blots were probed with antibodies directed against proteins associated with apoptosis (BAX, BCL2) and oxidative stress response (4HNE, NRF2, GPX4). Dashed line in the 4HNE Western blot (10w) shows area where non-relevant lanes were excised. Representative blots are shown (n = 4 mice per genotype). *F* and *G*, band intensities of proteins related to apoptosis (*B* and *C*) and oxidative stress response (*G*–*I*) were quantified using ImageJ software and normalized to the corresponding loading control. For 4HNE quantification bands of both molecular sizes were quantified and calculated as a mean. *D*, immunofluorescence staining of 8-oxo-guanosine (8-OxoG), a marker of DNA damage, was performed on testicular sections from WT and *Irp1*^*−/−*^ mice (n = 5 mice per group, 10 and 20 weeks old). Representative images are shown (scale bar: 50 μm). *E*, the number of 8-OxoG-positive cells (*green*) were quantified in round to slightly ovoid shaped seminiferous tubules (SFT) from five random images per animal using the ImageJ software. *J*, qPCR analysis of *Slc7a11* on testicular cDNA from testis of WT and *Irp*1^−/−^ mice aged 10 and 20 weeks. Statistical significance was determined using the unpaired student's *t* test (∗*p* < 0.05, ∗∗*p* < 0.01, ∗∗∗*p* < 0.001, n = 3–4 mice per genotype).

The underlying mechanism of hypospermatogenesis in IRP1-deficient testis was first analyzed by Western blot analysis of B-cell lymphoma 2 (BCL2) and Bcl2-associated X protein (BAX) as upstream apoptosis-related proteins. Quantification showed no significant difference (Fig. 6, *A*–*C*). Previous studies revealed that hypospermatogenesis can be a consequence of cell death triggered by high levels of ROS (22, 23). Given the observed downregulation of genes involved in DNA repair and upregulation of oxidative pathway genes (Fig. 4, *B* and *C*), ROS-induced DNA damage was assessed using 8-OxoG detection. Immunofluorescence labeling revealed a significantly higher number of 8-OxoG^+^ cells in *Irp*1^−/−^ testis compared to WT at both ages (Fig. 6, *D* and *E*). Of note, both spermatogonia and spermatocytes were most susceptible to ROS-induced DNA damage (Fig. 6, *D* and *E*). Besides DNA damage, high ROS levels also lead to lipid peroxidation. This was evidenced by an elevation of 4-hydroxynonenal (4HNE) in 10-week-old *Irp*1^−/−^ testis, which increased further at 20 weeks of age compared to WT (Fig. 6, *F* and *G*). Correspondingly, protein levels of nuclear factor erythroid 2-related factor 2 (NRF2), a transcription factor regulating apoptosis and lipid metabolism (including lipid peroxidation) (24), were significantly reduced in *Irp*1^−/−^ testis compared to WT at both ages tested (Fig. 6, *F* and *H*). Protein levels of glutathione peroxidase 4 (GPX4), an enzyme that controls lipid peroxidation (25), showed a trend towards downregulation, but did not reach statistical significance (Fig. 6, *F* and *I*). Moreover, cystine/glutamate antiporter solute carrier family 7 member 11 (SLC7A11) plays an important role in antioxidant defense by cysteine transport into cells, thus facilitating glutathione production and maintaining cell survival under conditions of oxidative stress (26). Using qRT-PCR analysis a significant downregulation of *Slc7a11* transcripts was visible in *Irp*1^−/−^ testis compared to wild-type (Fig. 6*J*), rendering IRP1-deficient testis more susceptible to oxidative stress.

## Discussion

Spermatogenesis is critically dependent on iron, serving as a vital co-factor for various enzymes involved in this process, making it essential for proper testicular function and male fertility. Various studies have shown that alterations in dietary iron concentration can lead to morphological changes in the testis, such as testicular atrophy, and impairment of spermatogenesis underscoring the importance of a tightly regulated iron balance in the testis (18, 27). Increased iron concentrations can trigger oxidative stress based on an imbalance of ROS and the antioxidant system where ROS levels predominate. In the testis, oxidative stress has several detrimental effects, such as DNA damage (base modifications, double-strand breaks) which impair the genetic integrity of germ cells (28). Simultaneously, oxidative stress not only generates harmful ROS but also decreases levels of antioxidants, such as superoxide dismutase (SOD1) and GPX4 in the testis (19, 28). The resulting damage can lead to increased cell death (*e.g.* apoptosis) causing hypospermatogenesis (18, 28, 29). Additionally, ROS can also lead to the formation of 4HNE, a major aldehyde product formed by peroxidation of ω-6-unsaturated fatty acids, regarded as a specific marker of lipid peroxidation that can cause several kinds of damage including dysfunction in the male germline (30, 31, 32, 33, 34).

Liu *et al.* (2022) showed that Slc7a11 is predominantly expressed in testicular somatic cells and is essential for maintaining cysteine/GSH homeostasis in Sertoli cells (26). Elevated cysteine levels during spermatogenesis are essential for protecting against oxidative damage from ROS. This puts SLC7A11 central in maintaining a redox balance in safeguarding germ cells. The observed substantial downregulation of *Slc7a11* transcripts in the testis of *Irp*1^−/−^ indicates a disrupted microenvironment that is permissive for increased ROS levels and associated damage as seen by the strong increase in 8-OxoG-positive germ cells compared to WT. Additionally, higher levels of 4HNE and the corresponding reduction of antioxidant factors GPX4 and NRF2 in *Irp*1^−/−^ testis further point to an imbalance in the oxidative stress system. Although data refer to a higher susceptibility of *Irp1*^*−/−*^ testis for oxidative stress, this does not seem to be related to higher concentrations of iron in the male gonad nor to a different distribution, as evidenced by ferritin localization.

Flow cytometry analysis in this study demonstrates a significant reduction of the number of primary spermatocytes (4N), while no changes were observed in cells with a chromosome content of 2N such as spermatogonia in germ-cell-enriched preparations. It is important to note that this method has some limitations, as it primarily differentiates cells based on their DNA content and may include contaminating Sertoli, Leydig, or peritubular cells which also are 2N.

In the next step, to obtain a comprehensive overview of the upregulated and downregulated genes, bulk RNA sequencing (RNA-seq) was performed. Whole genome transcriptomic analysis of *Irp*1^*−/−*^ testis demonstrated a down-regulation of cell cycle genes (mitosis, meiosis) and alterations in the repair mechanisms of DNA double-strand breaks. Many proteins involved in these processes require iron as a co-factor, particularly those associated with DNA replication or repair (35, 36). These data coincide with the reduced numbers of primary spermatocytes observed in our analysis, the germ cell type passing through meiosis, which in turn is propagated in lower numbers of haploid germ cells such as round and elongating spermatids. Together, these data indicate a defect in meiotic germ cells that either enter this process (very early primary spermatocytes) after a last mitotic division or in subsequent steps of meiosis (mid and late primary spermatocytes). Of note, an impairment of DNA repair or cell cycle in mitotic (spermatogonia) or early meiotic cells (primary spermatocytes)—as proposed by the transcriptomic analysis—potentiates the downstream effects, as one spermatogonia/primary spermatocyte entering meiosis results in the formation of four spermatids. Therefore, already a relatively mild loss of spermatogonia or primary spermatocytes could cause a more substantial reduction in numbers of more advanced germ cell types, *i.e.* hypospermatogenesis.

Recent studies have shown that functional impairment in the respiratory chain leads to a complete arrest of spermatogenesis from the primary spermatocytes onwards (37, 38). An alternative function of IRP1 when it contains a [4Fe-4S]-cluster is cytosolic aconitase, converting citrate to isocitrate in the cytoplasm, a function involved in providing intermediates for the Krebs cycle and other biosynthetic pathways crucial for cell proliferation and differentiation (17, 39). Li *et al.* demonstrated a downregulation of frataxin and IscU, two core components in the iron-sulfur cluster biogenesis machinery, in *Irp*1^−/−^ fibroblasts, thereby affecting the functionality of the respiratory chain (40, 41). Thus, the absence of cytosolic aconitase activity in *Irp*1^−/−^ testis could lead to metabolic insufficiency, limiting the availability of critical biosynthetic intermediates and energy required for spermatogenesis. Therefore, an additional explanation for the hypospermatogenesis observed in this study could be mitochondrial dysfunction due to the loss of cytosolic aconitase activity in *Irp*1^−/−^ testis negatively affecting germ cell production. Yet, further investigations are needed to confirm this hypothesis.

In summary, this study reveals a previously unrecognized function of IRP1 and provides clues on how IRP1 influences spermatogenesis that could guide tailored genetic analysis in men with this condition.

## Experimental procedures

### Animals and ethics statement

The *Irp*1^−/−^ (*Aco1*^*tm1Roua*^, MGI:3029223) (15) founder breeding pairs (bred on a C57Bl/6J background) were generously provided by Dr Tracey Rouault (Molecular Medicine Program, National Institute of Child Health and Human Development, National Institutes of Health, Bethesda, MD) to establish an experimental mouse colony at the animal facility of the Justus-Liebig-University (JLU) Giessen, Germany. Wild-type littermate controls were generated by interbreeding heterozygous mutant mice (*Irp*1^+/−^). This breeding strategy produced offspring with the following genotypes: wild-type (*Irp*1^+/+^), heterozygous (*Irp*1^+/−^), and homozygous mutant (*Irp*1^−/−^), in the expected Mendelian ratio of 1:2:1, respectively. All animal experiments were performed in accordance with the Technion regulations on animal welfare and German animal welfare law and were approved by the Technion Animal Ethics Committee, Haifa, Israel (*IL-135–09–19*) or had been declared to the Animal Welfare Officer of the JLU, Giessen, Germany (Registration No.: M_819). The methods used to euthanize the animals humanely were consistent with the recommendations of the AVMA Guidelines for the Euthanasia of Animals and performed by 5% isoflurane inhalation following cervical dislocation. Body weight and testis weight were recorded post-euthanasia.

### Human testis specimen

Bouin-fixed (12–24h) and paraffin-embedded human testicular biopsies from patients with azoospermia displaying intact spermatogenesis (nsp) or hypospermatogenesis (hypo) as spermatogenic disorder were provided by the Giessen Testicular Biopsy Repository and sectioned at 5 μm thickness. Specimens were categorized as described previously (42, 43). Written informed consent was obtained from all patients, and the study was approved by the ethics committee of the Medical Faculty of the Justus-Liebig-University Giessen (Ref. No. 26/11). All experiments were performed in accordance with the criteria set by the Declaration of Helsinki (44).

### Histology

Testes of postpubertal mice (10 and 20 weeks old) were either snap-frozen in liquid nitrogen for protein isolation or immediately immersion-fixed in Bouin's solution (Sigma, cat. no. HT10132-1L) for 6 h. Following fixation, tissues were dehydrated and paraffin-embedded, sectioned into 5 μm slices, and stained with hematoxylin and eosin (H&E) using standard protocols.

### RNA extraction, library preparation, and RNA sequencing (RNA-seq)

Total RNA was extracted from the whole testis of WT and *Irp*1^−/−^ mice by using the RNeasy Mini Kit from Qiagen (cat. Nr. 74,104, Hilden). RNA concentration and purity were measured using a NanoDrop 2000 (Thermo Fischer Scientific) and stored at −80 °C. All RNA samples had an RNA Integrity Number (RIN) of 8 or above, determined by Tapestation 4200. RNA sequencing libraries were generated and sequenced at the Institute for Lung Health (ILH)—Genomics and Bioinformatics—at the Justus-Liebig-University (JLU) Giessen (Germany). A total of 1000 ng of RNA per sample was used to enrich polyadenylated mRNA, followed by cDNA sequencing library preparation using the Illumina Stranded mRNA Prep Kit (Illumina) according to the manufacturer’s instructions. After library quality control by capillary electrophoresis (4200 TapeStation, Agilent), cDNA libraries were sequenced on the Illumina NovaSeq 6000 platform generating 50 bp paired-end reads.

### Bulk RNA-sequencing analysis

For demultiplexing and the subsequent FASTQ file generation, we used Illumina’s bcl2fastq (2.19.0.316). Primary processing of the sequencing reads, *i.e.*, quality control, filtering, trimming, read alignment, and generation of gene-specific count tables was performed using the nf-core 53 RNA-seq v3.7 bioinformatics pipeline (NEXTFLOW version 23.04.03). The mus musculus mm10 genome and gene annotation were used as downloaded from Illumina’s iGenome repository (https://support.illumina.com/sequencing/sequencing_software/igenome.html). The pipeline run was performed with standard parameters in docker mode. The resulting tables with raw read counts were imported into R where all down-stream processing was performed (R Core Team (2022) R: A Language and Environment for Statistical Computing. R Foundation for Statistical Computing, Vienna. URL https://www.R-project.org/) (version 4.3.2). Differentially expressed genes between *Irp*1^−/−^ and WT were identified using the DESeq2 package using the standard workflow outlined in the vignette, with an adjusted *p* value < 0.05 and |Log2fold change|> 1 as thresholds. These genes were used to create a PCA plot, a volcano plot, and a heatmap by using the ggplot2 package. Gene set enrichment analysis (GSEA) was performed as described by Subramanian *et al.* (2005) using the `*gsePathway*` function of the clusterProfiler package to identify Reactome terms associated with the mutant or wildtype phenotype (45, 46). We first ordered the differentially expressed genes according to ‘stat’, followed by visualization of the GSEA results. The ‘*gseaplot*’ function was used for visualization, with thresholds set at a *p*-value cutoff of 0.05 and *p*-value adjustment using the Benjamini-Hochberg (BH) method (45).

### Daily sperm production and sperm morphology

Daily sperm production (DSP) was determined as already described with small modifications (47, 48). Freshly collected testes were weighted, decapsulated, and homogenized in 1 ml DSP buffer (0.9% NaCl, 0.01% azide, 0.05% Trition-X-100) for 1 min at 30 Hz in a vibrating mill (Retsch MM400; Haan, Germany). After homogenization, intact sperm heads were counted in a Bürker counting chamber (0.0025 mm^2^, depth 0.100 mm). All homogenates were counted in duplicates, each in three different squares of the field of 16. To determine the DSP, an average value was calculated from the counted sperm (¥), and the following formula was used.

(1) ¥×50,000×(volumeofhomogenate[ml]×sampleweight[g])=n/homogenate

(2) n/homogenate÷sampleweight[g]=sperm/[g]testis

(3) sperm/[g]testis×totaltestisweight[g]=sperm/totaltestis

(4) sperm/totaltestis÷4.84=DSP/testis

Sperm morphology was assessed from spermatozoa obtained from dissected cauda epididymides. The cauda epididymidis was placed into a Krebs-Ringer solution (100 mM NaCl, 4.7 mM KCL, 1.2 mM KH_2_PO_4_, 1.2 mM MgSO_4_, 5.5 mM Glucose, 1.7 mM CaCl_2_, 20 mM HEPES, pH 7.0) to allow a sperm swim-out for 10 min at 37 °C, followed by a 10 min fixation step with 4% paraformaldehyde at RT with subsequent washing in PBS. Spermatozoa were air-dried on coverslips and analyzed under a light microscope using phase contrast. Representative images were captured with a Leica DMI 8 (Wetzlar, Germany) microscope and subsequently examined using ImageJ software (Fiji, version 2.1.0/1.53c). An equal number of five mice per genotype and age were used for analysis.

### Immunohistochemical staining on mice testes sections

Immunohistochemical staining was performed to identify changes in ferritin-H and -L distribution in testis. Antigen retrieval was done by boiling deparaffinized sections in 10 mM citrate buffer (pH 6.0), followed by blocking of endogenous peroxidase (3% H_2_O_2_ in ddH_2_O, 15 min). Sections were blocked with 3% BSA and 10% goat serum in tris-base-solution (TBS) for 1 h at RT and stained with antibodies against, ferritin-H (2 μg/ml, Abcam, ab65080, RRID:AB_10564857) and ferritin-L (2 μg/ml, Abcam, ab69090, RRID:AB_1523609) overnight at 4 °C in a humidified chamber. Subsequently, sections were incubated with respective secondary antibodies (goat anti-rabbit IgG conjugated with HRP, Cell Signaling Technology, 7074P2) for 1 h at RT. Detection was done using AEC detection (3-Amino-9-ethylcarbazol, Sigma Aldrich cat. Nr. AEC101), followed by hematoxylin counterstaining. Representative images were captured with a Leica Microscope DMi8, and image processing was carried out using ImageJ software. For semi-quantitative analysis, five random images of the testis sections were taken under the same settings. Followed by counting of all positive cells in round cross-sections of seminiferous tubules, an equal number of mice per genotype and age were used for analysis.

### SDS-PAGE and Western blot

Snap-frozen testis tissue (∼10 mg) was homogenized in RIPA-Buffer (50 mM Tris-HCL, pH 7.4, 150 mM NaCl, 0.5% deoxycholate, 0.1% SDS, 1% NP-40, AEBSF 0.1 M, 1 mM DTT) with phosphatase inhibitor (Roche, cat. No. 4906845001) and protease inhibitor cocktail (Roche, cat. No. 11836170001) followed by incubation on ice for 30 min. Protein concentration in supernatants of centrifuged samples (20,000*g*, 30 min, 4 °C) was measured by the BCA protein assay kit (Sigma-Aldrich, cat. No. BCA1-1KT).

Equal amounts of protein (50–100 μg) were separated on 10 to 15% SDS-PAGE and transferred to PVDF membranes (Merck Millipore, cat. no. IPVH00010). Membranes were blocked with blocking solution (Tris-buffered saline with 0.1% Tween-20 supplemented with milk powder) for 1 h at RT, before being incubated with primary antibodies (see Table 1) overnight at 4 °C. Membranes were washed and incubated with horseradish peroxidase-conjugated secondary IgG antibodies (α-rabbit, Abcam, ab97200 RRID:AB_10679899; α-mouse, DanyelBiotech, NXA931, RRID:AB_772209) for 1 h at RT. Signals were visualized using ECL (Millipore Corporation) and analyzed by densitometry (Fusion FX, Witec AG). Signal intensity was quantified using ImageJ software (Fiji software, version 2.1.0/1.53c).

**Table 1:** Antibodies used for Western Blot

Antibodies	RRID number	Dilution	Source	Supplier
Ferritin-L	RRID:AB_1523609	1:1000	Rabbit	Abcam, Ab69090, Cambridge, UK
Ferritin-H	RRID:AB_10564857	1:1000	Rabbit	Abcam, Ab65080, Cambridge, UK
Ferritin-H	RRID:AB_11217441	1:1000	Rabbit	Cell Signaling Technology, 4393S, Cambridge, UK
Ferroportin/SLC40A1	RRID:AB_1660490	1:1000	Rabbit	Novus Biologicals, NBP1-21502, Wiesbaden, Germany
Transferrin Receptor	RRID:AB_10673794	1:1000	Rabbit	Abcam, ab84036, Cambridge, UK
IRP1	RRID:AB_11024630	1:1000	Rabbit	Novus Biologicals, NBP1-87677, Wiesbaden, Germany
IRP2		1:1000	Rabbit	Novus Biologicals, NBP100–1798–1, Wiesbaden, Germany
NRF2 (C-20)	RRID:AB_2108502	1:500	Rabbit	Santa Cruz, sc-722, Dallas, Texas, USA
GPX4	RRID:AB_10973901	1:2000	Rabbit	Abcam, ab125066, Cambridge, UK
4HNE	RRID:AB_664165	1:1000	Mouse	R&D Systems; MAB3249, Wiesbaden, Germany
BAX (N-20)	RRID:AB_2227995	1:500	Rabbit	Santa Cruz, sc-493, Dallas, Texas, USA
BCL2 (N-19)	RRID:AB_2064290	1:250	Rabbit	Santa Cruz, sc-492, Dallas, Texas, USA
Beta Actin	RRID:AB_476744	1:3000	Mouse	Sigma Aldrich, A5441, Taufkirchen, Germany

### Ferrozine assay

Total nonheme iron in testis and liver samples was measured by colorimetric ferrozine-based assays as previously described (49). Briefly, 11 μl concentrated HCl (11.6 M) was added to 50 μl of homogenized tissue samples, heated at 95 °C for 20 min and centrifugated at 13,000 rpm for 10 min. Ascorbate was added to the supernatant for 2 min, to reduce the Fe^3+^ into Fe^2+^, followed by the addition of ferrozine and saturated ammonium acetate (NH4Ac). Absorbance was directly measured at 570 nm (BERTHOLD microplate reader).

### Determination of DNA content in germ cells by flow cytometry

Decapsulated testes were transferred to a centrifuge tube (50 ml) in 3 ml Dulbecco's Modified Eagle Medium (DMEM) (Gibco, Thermo Fisher Scientific) containing 1 mg/ml collagenase A (Roche, cat. No. 10103578001) and 1 mg/ml DNase I (Roche, cat. No. 10104159001) and were incubated under constant agitation in a water bath (120 rpm) at 34 °C for 15 min. The resulting tubule fragments were allowed to settle in the tubes for 2 min and then used for another digestion round in 2.5 ml DMEM with collagenase A, 0.004 mg/ml of DNase I, and 1 mg/ml of trypsin (50 mg/ml in 1 mM HCL, Sigma-Aldrich, cat. No. SLBR7267V). The final digest involved adding 0.6 mg/ml of trypsin and 0.004 mg/ml of DNase I, with another round of agitation. To inhibit trypsin activity, 10% of FCS (Gibco, ThermoFischer Scientific, cat. No. 10500064) was added. This procedure enriches germ cells. The cell suspension was then passed through a 40 μm nylon cell strainer (Corning) and centrifuged at 1500 rpm for 10 min at 4 °C. The pellet was resuspended in DPBS and cells were counted. A total 1 × 10^6^ cells resuspended in 100 μl were used for subsequent steps. Cells were first permeabilized on ice with 90% methanol for 30 min, washed and resuspended in 500 μl MACS-Quant buffer, containing 5 μg/ml propidium iodide (BioLegend, cat. 421301) and incubated 30 min at 4 °C. Finally, the DNA content was quantified using the MACSQuant Analyzer 10 (Miltenyi Biotec), and the results were evaluated using FlowJo version 10.8.0 software (FlowJo LLC).

### Immunofluorescence on human biopsies and mice testes sections

For human and mouse testes, antigen retrieval was performed as described above. Sections were blocked with either 3% BSA in PBS or 1% BSA and 10% normal goat serum in 1× TBS + 0.5% Triton X-100 for 1 h. Human testes biopsies were stained with ferritin-H (2 μg/ml, Abcam, ab65080, RRID:AB_10564857) and ferritin-L (2 μg/ml, Abcam, ab69090, RRID:AB_1523609) antibodies overnight at 4 °C. For mouse testes sections, the primary antibodies used were anti-8-oxoG DNA lesion (10 μg/ml, Santa Cruz Biotechnology, Cat# sc-130914, RRID:AB_2009328) and anti-SCYP3 (1 μg/ml, Abcam, Cat# ab97672, RRID:AB_10678841). Both were incubated overnight at 4 °C. Followed by incubation with the secondary antibodies (goat anti-mouse IgG (H + L) cross absorbed secondary antibody Alexa Fluor488 (1 μg/ml, Invitrogen, Cat# A-11001, RRID:AB_2534069) and goat anti-rabbit IgG (H + L) cross absorbed secondary antibody Alexa Fluor488 (1 μg/ml, Invitrogen, Cat# A-11008, RRID:AB_143165)) for 1 h at RT. Mounted specimens were examined using a Zeiss LSM710 Confocal Microscope (Oberkochen). Image processing was carried out using ImageJ software. For the semi-quantitative analysis, five random images of testicular sections were taken under the same settings, followed by counting all positive cells in round cross-sections of seminiferous tubules. The analysis included five mice for each genotype and age.

### qRT-PCR

RNA was isolated as described above. Subsequently, 1 μg of RNA was reverse-transcribed to cDNA. Quantitative PCR was carried out on a CFX96 Touch thermal cycler machine (Bio-Rad) using iTaq Universal SYBRGreen SuperMix (Bio-Rad) and primers specific for *Slc7a11* (Table 2). The relative accumulation level of each mRNA was normalized to *18S* rRNA as a reference, and the comparative Ct method (ΔΔCt method) was employed for the data analysis.

**Table 2:** Primer sequence for RT-qPCR

Gene	Forward	Reverse
*Slc7a11*	GCTGGTGTGTAATGATAGGGC	CCCCTTTGCTATCACCGACT
*18sRNA*	TACCACATCCAAGGAAGGCAGCA	TGGAATTACCGCGGCTGCTGGCA

### Statistics

Statistical analyses were performed using the GraphPad Prism 8 software (GraphPad Software). To check for normal distribution, data were first transformed to log values. Values were used to create QQ plots and to perform Kolmogorov-Smirnov and Shapiro-Wilk tests. The samples were statistically analyzed using the unpaired student's *t* test. All data are represented as mean ± SEM, and significance levels were denoted as ns>0.05, ∗*p* < 0.05, ∗∗*p* < 0.01, and ∗∗∗*p* < 0.001.

## Data availability

The bulk RNA sequence data have been uploaded to Gene Expression Omnibus (GEO; GSE273580). Additional data and the used R code are available from the corresponding author.

## Supporting information

This article contains supporting information.

## Conflict of interest

The authors declare that they have no conflicts of interest with the contents of this article.
