# Supplementary material for: Iron regulatory protein 1-deficient mice exhibit hypospermatogenesis
Source: J Biol Chem. 2024 Dec 10;301(1):108067. doi: 10.1016/j.jbc.2024.108067 (PMC11758943; doi:10.1016/j.jbc.2024.108067)
Supplement: Supporting information [file mmc1.docx]

**Supporting information**

**Iron regulatory protein 1 deficient mice exhibit hypospermatogenesis**

Aileen Harrer*^1,2^, Niraj Ghatpande*^3^, Tiziana Grimaldini^1,2^, Daniela Fietz^2,4^, Vishnu Kumar^1,2^, Christiane Pleuger^1,2^, Monika Fijak^1,2^, Dankward T. Föppl^1,2^, Lennart P. Rynio^1,2^, Hans‑Christian Schuppe^2,5^, Adrian Pilatz^2,5^, Marek Bartkuhn^6^, Tara Procida‑Kowalski^6^, Noga Guttmann-Raviv^3^, Sudhanshu Bhushan^1,2^, Esther G. Meyron-Holtz^3, #^ and Andreas Meinhardt^1,2, #^

**Supporting Figures:**

**
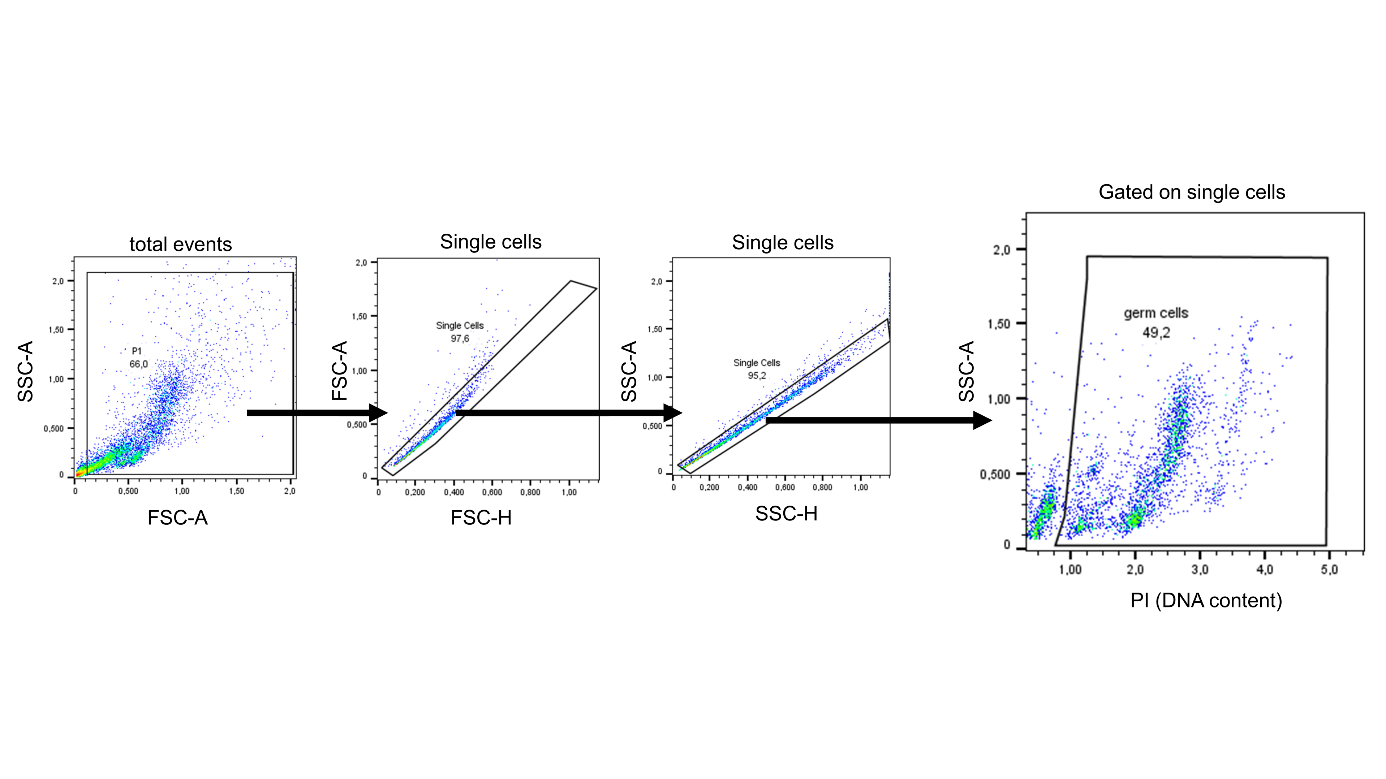
**

**Supporting Figure 1:** Gating strategy used to identify different DNA content in germ cell enriched cell populations in flow cytometry analysis of testis samples from 10- and 20-week-old mice is shown. Single live cells (based on forward and side scatter) were initially gated, followed by selection of propidium iodide (PI)-positive cells (PI^+^) representing single testicular cell suspension.
